# Rare pseudo-chediak-higashi inclusions in a patient with disseminated diffuse large B cell lymphoma

**DOI:** 10.1007/s44313-024-00013-x

**Published:** 2024-03-25

**Authors:** Can Yan, Zenghui Fang, Jinlin Liu

**Affiliations:** 1 Department of Clinical Laboratory, Jinhua Municipal Central Hospital, Jinhua Hospital of Zhejiang University, Jinhua, China; 2 Department of Clinical Laboratory, South China Hospital, Medical School, Shenzhen University, 1 Fuxin Road, Shenzhen, 518111 China

**Keywords:** Pseudo-chediak-higashi inclusions, Disseminated diffuse large B cell lymphoma

A 45-year-old male was admitted to the hospital with complaints of malaise, chest tightness, and abdominal distension persisting for two weeks. Physical examination revealed pleural and peritoneal effusions. Initial hematological results showed a hemoglobin level of 74 g/L and a platelet count of 45 × 10^9^/L. A blood smear showed peripheral blood involvement (10%) through lymphoma-like cells, and bone marrow aspiration showed involvement of these lymphoma-like cells (90%) (Fig. 1A). These cells exhibited nuclear indentation and eccentric nuclei. Notably, a few giant pink-purple inclusions (chédiak-higashi-like inclusions) were observed in the cytoplasm of these lymphoma-like cells (Fig. 1A). These inclusions were situated adjacent to the nucleus and displayed variations in size, with negative myeloperoxidase staining (Fig. 1B) and positive Schiff periodic acid-shift staining (Fig. 1C). Furthermore, cytological examination of pleural and peritoneal fluid revealed infiltration by the same cells harboring pseudo-chediak higashi inclusions. Flow cytometry analysis demonstrated positivity for FMC7, CD10, CD19, CD20, CD22, kappa, HLA-DR, and CD200. Cytogenetic studies showed 46, xy, dup (12) (q13 q21), t(14:18), (q32; q21.3) [4] /46, xy [10]. Genetic testing revealed KMT2D p.Q1029*, KMT2D p.P4865Lfs*5, and EZH2 p.Y646S using the Illumina next-generation sequencing platform. Consequently, a diagnosis of disseminated EZB type diffuse large B-cell lymphoma (EZB-DLBCL) was established. After the diagnosis of disseminated DLBCL, the patient underwent four cycles of R-CHOP and received intrathecal injections (cytarabine, methotrexate, and dexamethasone) once. The tumor burden significantly decreased, as evidenced by PET-CT, and stem cell transplantation was considered before manuscript submission.

**Fig. 1:**
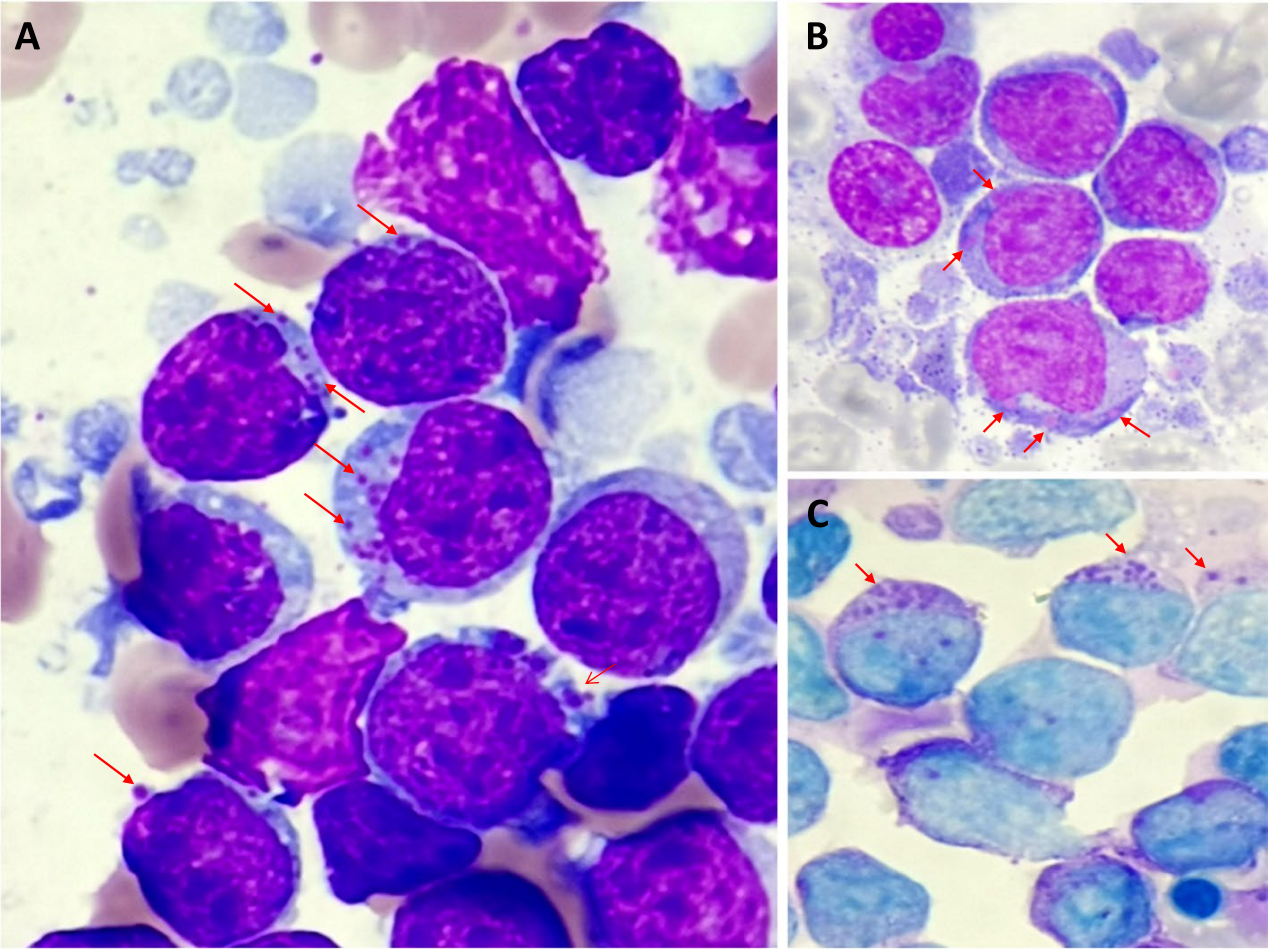
**A** Few giant pink-purple inclusions (chédiak–higashi-like inclusions) were found in the cytoplasm of lymphoma cells in bone marrow aspiration (red arrow, Wright–Giemsa staining, × 1000); a negative myeloperoxidase staining (**B** red arrow), and a positive schiff periodic acid staining (**C** red arrow) were observed for these chédiak–higashi-like inclusions

Pseudo-Chediak-Higashi inclusions are occasionally reported in various hematological malignancies such as acute myeloid leukemia, acute lymphoblastic leukemia, myelodysplastic syndrome, chronic myeloid leukemia, and mixed-phenotype acute leukemia, referred to as pseudo-Chédiak-Higashi anomalies, with unclear pathogenesis [1, 2]. These inclusions are typically not observed in patients with lymphoma, making the presence of rare pseudo-Chediak-Higashi inclusion in a disseminated EZB-DLBCL patient exceptional.

## Data Availability

No datasets were generated or analysed during the current study.
